# Supplementary figures and images for: Prediction of active ingredients in Salvia miltiorrhiza Bunge. based on soil elements and artificial neural network
Source: PeerJ. 2022 Jan 18;10:e12726. doi: 10.7717/peerj.12726 (PMC8781443; doi:10.7717/peerj.12726)

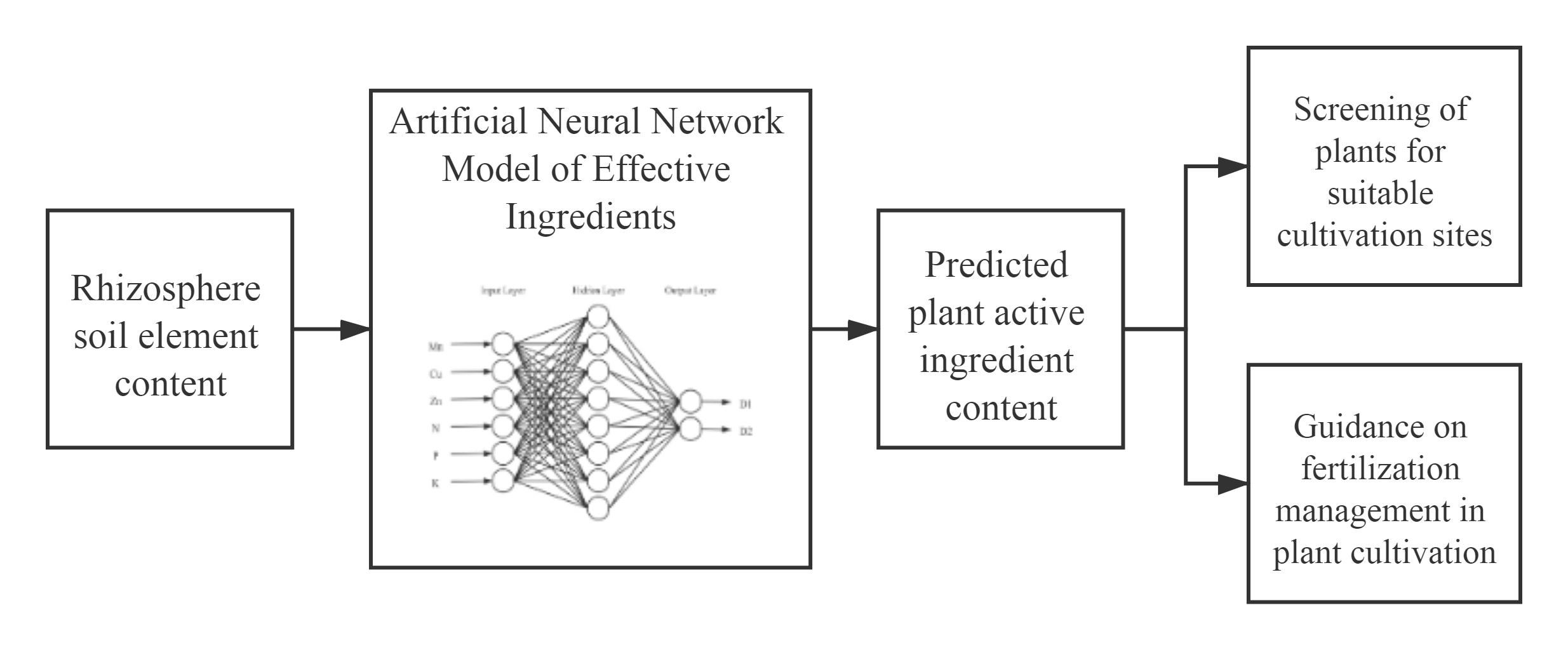

Supplement: Supplemental Information 3 [file peerj-10-12726-s003.png]
